# Health worker preferences for performance-based payment schemes in a rural health district in Burkina Faso

**DOI:** 10.3402/gha.v9.29103

**Published:** 2016-01-05

**Authors:** Maurice Yé, Eric Diboulo, Moubassira Kagoné, Ali Sié, Rainer Sauerborn, Svetla Loukanova

**Affiliations:** 1 Centre de Recherche en Santé de Nouna, Nouna, Burkina Faso; 2 Institute of Public Health, University of Heidelberg, Heidelberg, Germany; 3 Department of General Practice and Health Services Research, University of Heidelberg Hospital, Heidelberg, Germany

**Keywords:** Burkina Faso, performance-based incentive, pay for performance, performance-based financing, maternal health, child health, motivation, healthcare providers

## Abstract

**Background:**

One promising way to improve the motivation of healthcare providers and the quality of healthcare services is performance-based incentives (PBIs) also referred as performance-based financing. Our study aims to explore healthcare providers’ preferences for an incentive scheme based on local resources, which aimed at improving the quality of maternal and child health care in the Nouna Health District.

**Design:**

A qualitative and quantitative survey was carried out in 2010 involving 94 healthcare providers within 34 health facilities. In addition, in-depth interviews involving a total of 33 key informants were conducted at health facility levels.

**Results:**

Overall, 85% of health workers were in favour of an incentive scheme based on the health district's own financial resources (95% CI: [71.91; 88.08]). Most health workers (95 and 96%) expressed a preference for financial incentives (95% CI: [66.64; 85.36]) and team-based incentives (95% CI: [67.78; 86.22]), respectively. The suggested performance indicators were those linked to antenatal care services, prevention of mother-to-child human immunodeficiency virus transmission, neonatal care, and immunization.

**Conclusions:**

The early involvement of health workers and other stakeholders in designing an incentive scheme proved to be valuable. It ensured their effective participation in the process and overall acceptance of the scheme at the end. This study is an important contribution towards the designing of effective PBI schemes.

## Introduction

Improving maternal and child health remains the most critical challenge of the Millennium Development Goals (MDGs). Sub-Saharan Africa countries bear the largest burden with an average maternal mortality ratio (MMR) of 500 deaths per 100,000 live births (1). In Burkina Faso, MMR and neonatal mortality remain unacceptably high at 341 per 100,000 live births and 28 per 1,000 live births, respectively (2). Burkina Faso set out to achieve the goal of MMR of 121 per 100,000 live births by 2015. With an annual progress of only 3.5% made so far, the country struggles to meet the MDGs targets in the backdrop of the poor quality of care and low health worker motivation (3).

Among factors affecting the quality of health care, low motivation of health workers has been consistently reported. According to Franco's theory of motivation (4), health worker performance is a function of *competence, motivation, and factors including resource availability and other supports*.

However, over the past years this low quality of care has been framed mainly in terms of low resources (5–10). The response of many governments and donors has been targeted at purchasing inputs, but this has not resulted in substantial improvements in the quality of care either (9, 11).

A large body of evidence has been accumulated describing the low quality of health care in low- and middle-income countries (LMIC) (12, 13).

Results based financing (RBF) is defined as any programme that rewards the delivery of one or more outputs or outcomes by one or more incentives, financial or otherwise, upon verification that the results agreed upon have actually been delivered (14). The strategy has been promoted by a number of donors, including the World Bank, the World Health Organization (WHO), the Global Alliance for Vaccines and Immunization (GAVI), the US Agency for International Development (USAID), non-governmental organizations (NGOs), and faith-based organization (15, 16). In line with this, performance-based incentive (PBI), also known as performance-based financing (PBF), is seen as a promising strategy to improve the motivation and quality of care by a majority of the development partners (17–23).

According to the World Bank's recent statistics (24), PBF mechanisms have been introduced or explored in more than 30 LMIC in the health sector with the objective of improving performance of health service providers. Rwanda and Burundi, with the support of development donors, were at the forefront of the implementation of these methods nationwide in Africa (18, 19, 23, 25).

Most studies (18–21, 26) on PBF during the past decade provided evidence that it did indeed positively influence the quality of care. In Burundi, the probability of a child being fully vaccinated increased by 4 percentage points after the introduction of PBF (25). In Cambodia, within a PBF scheme supported by the GAVI fund in 2007 (27), the quantity and quality of maternal and child care services were significantly boosted.

In Battagram district in Pakistan (16), a PBI scheme whereby all government-employed health facility workers were entitled to receive an additional 20–35% of their salary upon achievement of prescribed targets was implemented. This scheme led to a 150% increase of skilled birth attendants and an 89% increase in immunization coverage.

Despite the above-mentioned advantages associated with the PBI scheme, disadvantages have also been noted with suggestion that it is not sustainable, it will not have a pro-poor effect, or it may create perverse incentive (15, 28–30). Wynia (31) suggests that payment systems can create incentives for unethical behaviour by setting the physician's pecuniary interests in opposition to high quality care and that adding an extrinsic financial incentive might undermine, or ‘crowd out’, intrinsic motivation.

Previous empirical studies (15, 32) suggest that unintended consequences and perverse effects are most likely to happen for both financial and non-financial incentive schemes. In Rwanda (18), it was found that rewards for good performance were most effective in improving outcomes that appear to have the highest marginal return or require the least effort. Furthermore, it was argued that among contracted outcomes, providers may also allocate effort to those that yield the largest (net) marginal return (15, 29, 30, 33). This was labelled as ‘Gaming’, effects observed in the health sector and includes falsification of data and oversupply of targeted services (15, 34, 35). Thus, the benefits of PBF remain partly inconclusive. The World Bank notes that schemes based on endogenous funding systems are scarce and should be promoted (16, 34, 36).

In the Nouna Health District (NHD), a supply-side incentive model was opted. Supply-side incentives are typically targeted at health managers, health institutions, and/or their staff, and tied to the achievement of predefined performance indicators that are set out in a performance contract (37).

In Burkina Faso, PBI schemes are very new. At the time of the study in 2010, there was no ongoing PBI intervention in the country.

Our study aimed to explore in a participatory approach which centrally involves healthcare providers, the most appropriate and locally adapted PBI schemes.

To develop such a scheme, which will on one hand be designed through a participatory process involving healthcare providers, and on the other hand will be primarily and financially supported by the district's own resources, it was important to understand providers’ preferences about the planned incentive schemes and the existing revenue sources that could support these schemes.

The adopted participatory process was found to be unique from the literature as it gives high priority to the sustainability of the proposed schemes. However, as noted by several authors (25, 34, 36), although the World Bank has initiated several PBF pilots across Africa with associated impact evaluations, schemes based on endogenous funding systems are scarce.

Our study was built on local contextual factors as well as endogenous funding sources to propose a locally adapted incentive scheme. It was also meant to be a stepping stone for implementation of the incentive scheme for health workers in rural districts which can be an alternative to externally funded PBFs. Key terms and definitions are provided in the Supplementary Table 1.

## Design of the provider-developed scheme

The planned PBI scheme placed its emphasis on locally generated resources. Traditionally, health facilities raise revenue mainly through user fees and the sale of drugs and other pharmaceuticals. These funds are used for personnel revenue accrued to nurses. In addition, health workers often receive substantial per diem payments from participation in workshops and seminars. These are generally organized by the Ministry of Health, by national and international NGO's funded research projects, or by multilaterals such as United National Children's Fund (UNICEF), The World Bank, etc. (38). These constitute a substantial part of the health staff's income, sometimes surpassing their salary.

The proposed incentive scheme suggests a locally adapted and more sustainable funding system, which consists of pooling a portion of that money in a district fund to pay incentives. Ridde (38) describes various negative aspects of per diem generated income, which includes overplanning of actions around the primary goal of acquiring per diems rather than of effecting changes among the public targets by their intervention. Other negatives include a decrease in motivation for activities without per diems, tendency to prefer workshops with per diem over those without them, etc. However, it has become a substantial part of income for health staff, sometimes even surpassing their basic salaries.

**Fig. 1:**
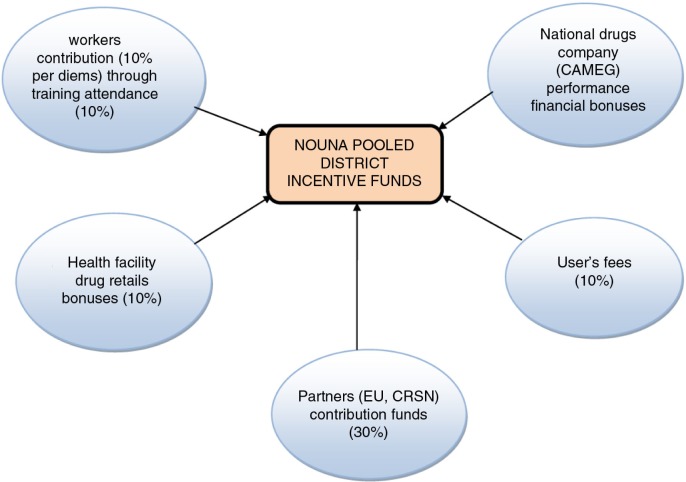
Source of incentive funds. EU: European Union; CRSN: Centre de Recherche en Santé de Nouna; CAMEG: Centrale d'achat des Médicaments Essentiels Génériques.

The incentive scheme developed in this study together with health staff involves the pooling of a portion of per diem money into a district fund (Nouna Pooled Incentive Funds) alongside other sources to pay out the final incentives to the staff (Fig. 1).

## Description of health financing mechanism in place and relevant policies

The health financing system in the NHD is an input-based financing system rolled out by the Central Ministry of Health in the form of financial support to each district contingent upon an approved annual action plan (3). These funds come from national budgets, development partner's funds, and other district revenues. A national body, *Programme d'Appui aux Development Sanitaire*, is the main recipient of donors’ funds directed towards supporting district health activities. In addition, NHD benefits from different funding sources mainly from NGOs such as ‘Foundation Terre des Hommes, Germany, University of Heidelberg, Germany, community-based health insurance (CBHI) contracting, and faith-based organizations’. This funding mechanism was tailored for purchasing equipment, conducting outreach activities, and supporting operational and other running costs in the health facilities. Within this health system financing strategy, the district health management team (DHMT) receives funds to purchase inputs and organize local training sessions. Experience showed that the lack of accountability of managers often led to misspending of these funds with dramatic consequences such as frequent shortages in drug procurement. At the time of the study, there was an ongoing CBHI scheme in Nouna. This CBHI scheme used a third-party payment mechanism to finance care provided to the enrolees (39). Within this payment mechanism, primary- and secondary-care facilities, which had contracted with the scheme, were paid on an annual capitation basis.

## Performance-based incentive implementation phases

This study is part of a broader project aimed at improving quality of prenatal and maternal care ‘Quality of Maternal and Child Health care (QUALMAT)’ funded by the European Union (EU) with two key interventions: 1) PBI to increase health workers’ motivation in providing high quality of care and 2) a computer-assisted clinical decision support system aimed at helping providers comply with recommended standards of care (40).

**Fig. 2:**
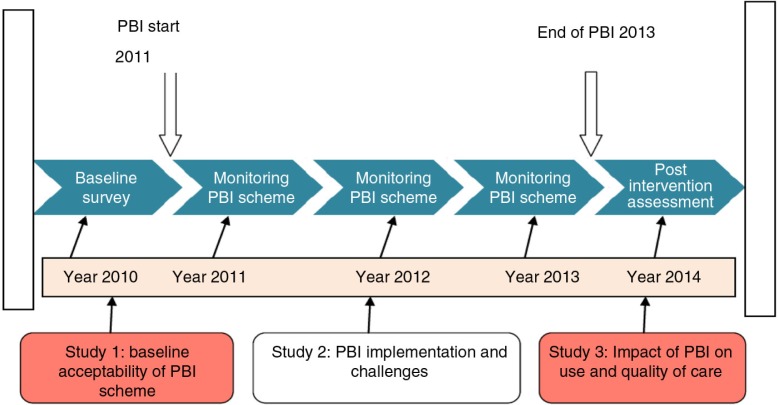
Sequences of studies within the PBI project period 2010–2014.

The study was designed to collect both baseline and post intervention data and was implemented from 2010 to 2014 in two different health districts, Nouna and Solenzo. Nouna district acted as the intervention district, whereas Solenzo was the control. The current study is based on the baseline data gathered prior to the intervention in Nouna. Figure 2 depicts the different phases involved in the intervention.

## Methods

### Study setting

The study was conducted in the rural NHD, in Burkina Faso, covering a population of approximately 312,000 inhabitants served by 34 health facilities. These include 33 dispensaries, which are staffed by nurses, midwives, and nursing-aids and a 100-bed district hospital located in Nouna town (41).

### Study design

A cross-sectional mixed survey design was used to collect both qualitative and quantitative data. The mixed design was adopted as it best fits our study objectives and allowed a thorough understanding of both qualitative and quantitative factors associated with workers’ decision to participate or refrain from participation in a locally constructed PBI scheme. Data collection was conducted from May to July 2010.

### Population and sampling strategy

#### Quantitative study

Maternal and child health care (MCH) providers, including health facility managers who had been trained and were in that position for at least one year, were eligible for the study. One hundred twenty-six health workers in the whole district met these criteria. The sample size was calculated based on 5% margin of error and 95% confidence level, which led to the inclusion of 94 health workers in the study.

#### Qualitative study

A sample of 33 (35%) health workers out of the 94, identified and representing the different categories of health staff, was retained for in-depth interviews to elicit the views of MCH providers about their preferences for the proposed PBI scheme. In particular, respondents from both sexes were included consisting of nurses (20), midwives (08), and health managers (05).

#### Data collection

We developed a survey questionnaire, which was pretested on respondents. The survey questionnaire included five sections: 1) socio-demographic characteristics of respondents; 2) acceptance of a PBI scheme; 3) preference for financial versus non-financial incentives; 4) level of financial contribution into the district funds; and 5) suggested performance indicators.

For the qualitative component of the study, in-depth interviews were conducted using a semi-structured interview guide to capture workers’ opinions about the scheme. Duration of the in-depth interview was about 90 min on average. Data were collected by senior field workers with proven experience in conducting qualitative interviews.

Fieldworkers were trained for 3 days to get them familiarized with data collection tools and field procedures.

The in-depth interviews were conducted in French, the official language spoken by health workers. All the interviews were tape-recorded and transcribed.

All data collected in the field were checked thoroughly for consistency.

Quantitative data were processed with Stata software.

#### Data analysis

Statistical analyses of the quantitative data were performed using STATA version 11.2. Parameters were estimated with 95% confidence level.

In addition, Nvivo.8 QSR was used to organize and code the qualitative data. A summary of coding methodology, which was extensively described elsewhere (42), is provided in Table 1.

**Table 1:** Coding framework according to main themes and sub-themes

Main themes	Sub-themes	Sub-themes
Financial incentive (all cited aspects related to monetary)	Salary increase – Pension – Tax relief – Upgrade salary Bonuses – Overtime payment – Extra time payment – Hardship payment Per diems – Money during training – Money during workshop Position allowance – Management fees – Equipment fees Phone allowance – Fees for in duty call – Allowance for management	Loans – Payment of school fees – Fees for housing – Fees for vehicle and motorbike Risk allowance – Extra duty fees – Pay insurance – Uniform allowance Rural allowance – Remote area fees – Transport fees – Outreach allowance Support in retirement – Paid leave – Subsidize health care
Non-financial incentive (include activities related to non- monetary support)	Trainings – Regular trainings – Seminar – On job trainings – Refresher courses – Graduate trainings Supportive coaching – Supervision – Feed-back – Problem solving – Regular moving Accommodation – Improve housing condition – Ensure transport for workers	Working condition – Equipment – Logistic – Adequate work place – Work flexibility – Security at working place Appreciation – Verbal appreciation – Letter of appreciation – Awards – Prize – Timely promotion
Incentive type (team-based or individual-based)	Team incentive Individual incentive	
Performance indicators (domain to derive indicators for facilities rewards	Maternal health – Antenatal care indicators – Prevention of mother to child HIV/AIDS – Delivery care – Post-partum care – Family planning	Neonatal – Immunization – Newborn care at birth – Prophylaxis
Frequency of incentive	Monthly Quarterly Yearly	Allocate money according to specified time

#### Ethical approval

This study was part of the QUALMAT project *Improvement of pre-natal and maternal health care*, funded by EU grant 2009–2014. Grant agreement number 22982.

The protocol received approval from institutional review boards in both countries (Centre de Recherche en Santé de Nouna (CRSN), Burkina Faso, and University of Heidelberg, Germany).

## Results

### Characteristics of the respondents

Of the 94 respondents, 28% were female nurses and midwives, representing 30% of all health care providers as shown in Table 2. In addition, qualitative study with some participants comprised 25 males and 8 females, of which 20 were nurses, 8 were midwives, and 5 were health facility managers. Table 2 shows the detailed characteristics of the respondents.

**Table 2:** Socio-professional characteristics of respondents (*n*=94)

	Distribution (*n*=94)	
	
Variables	*N*	%
Characteristics of respondents		
Age		
20–29	23	24.4
30–39	49	52.1
40–49	22	23.4
Mean age (34.5) years		
Sex		
Male	68	72
Female	26	28
Professional category		
Medical doctor	1	1
Nurse	38	41
Midwife	20	21
Nurse assistant	35	37
Working post		
District manager	5	5.3
Facility manager	34	36.1
Other workers	55	58.5
Years of experience		
1–2	23	24.46
3–5	49	52.12
≥6	22	23.4
Total	94	100

### Acceptability and preferences for PBI scheme

Out of 94 respondents who participated, 85% (80 out of 94) were in favour of an incentive scheme funded by health facilities (95% CI: [71.9; 88.08], *p*<0.001). Moreover, a majority of healthcare providers, 95% (76 out of 80) opted for financial incentives. The results were statistically significant (95% CI: [66.64; 85.36], *p*<0.001).

In addition to receiving cash, some respondents (four) would prefer to see a non-financial incentive also included in the system.

However, 15% of the respondents (14 out of 94) were undecided about the overall incentive scheme.

### Team-based versus individual-based incentive

As to whether incentive should be given to the entire team or to individuals, most respondents, 96% (77 out of 80), were in favour of incentives being provided to the entire team. The result was statistically significant (95% CI [67.78; 88.22], *p*<0.001).

### PBI acceptance by professional category

The acceptability rate for the incentive system was 89.7% for health facility managers and 81% for other workers. There was no statistically significant difference between the two categories (*p*=0.43). Acceptability rate for financial incentive was 97% among health facility managers and 93% for other workers. The difference was not significant (*p*=0.47).

### Suggested levels of financial contribution

The most preferred level of financial contribution suggested by respondents was 10 and 20% of health facility generated income with, respectively, 46% of respondents (37 out of 80) and 24% of respondents (19 out of 80) as in Supplementary Table 2.

### Results from qualitative data analysis

The qualitative study involved 33 health providers and was aimed at understanding their perception of the proposed scheme.

#### Health workers’ views and preference of the locally funded PBI scheme

Most respondents were not aware of the overall PBI scheme. However, the proposed PBI scheme was much appreciated as a way to reward well-performing health workers. Among the perceived advantages, the promotion of solidarity among colleagues was mentioned. The improvement of productivity and performance of healthcare providers was also cited. In contrast, some disadvantages such as sustainability of the system was noted. Few respondents argued about the long-term sustainability of locally generated funds. The issue of transparency in the management of such funds was mentioned:

This incentive strategy is a good idea; however, this system should rely on the subsidies from the government rather than the district funds for sustainability. (ST, male midwife, 37 year, PHC)

Another respondent also shared this viewpoint and pledged for better working conditions

I think managers should pay attention to our working conditions by providing suitable environment, more equipment. (Male nurse, 23 years, DRA, PHC)

#### Preference for financial incentive

Preference for financial incentive was clearly expressed by the majority of respondents. As one respondent said:

Providing financial incentives to workers based on their performance can be motivating and can improve the quality of care. It builds also a more competitive team. (Male nurse, 36 years, BRS, PHC)

One maternal care provider also shared this viewpoint:

I prefer also to get money, as here we are working very hard with a little salary. Receiving extra money is motivating and will surely help us improve our living conditions. (Female midwife, 32 years, DBS, maternity ward)

Among the perceived advantages mentioned: 1) financial incentive can be a booster for workers’ performance; 2) can lead to immediate results; and 3) can be a top-up to low salaries. Another participant argued in favour of financial incentive:

We are embarked in a French system where workers who come for training are usually given money. When you leave your setting for training, you have to look for accommodation, food, and other incidentals, financial incentive is very important. (Male, health facility manager, 34 years, BBK, PHC)

Some respondents argued that financial incentives cannot last longer or be sustained over time. For some, it is difficult to determine the level of money that can lead to a high performance.

#### Preference for non-financial incentive

A minority of the respondents, in addition to getting financial incentives, would have liked to see a non-financial incentive such as improved working conditions and upgrading facility equipment. As it was noted by one respondent:

In addition to giving money, providing sometimes non financial rewards, working material and suitable environment, can motivate us to work hard. (Male nurse facility manager, 39 years, PHC)

Another participant pointed out:

I suggest first of all that the manager look for strategies to improve staff working conditions, i.e. equipment, training opportunities and career development as money cannot overcome all problems. (Nurse, facility manager, 35 years, DBO, PHC)

In terms of perceived advantages by the pros for non-financial incentive, the following arguments were raised:

By recognizing workers through letters or verbal appreciation, this can motivate better; motivation is not only financial, the other aspects are very useful to consider. (Female midwife, ward manager, 27 years, PHC)

There were also perceived disadvantages associated with non-financial incentive as stated by one participant:

In the context of low salary, providing only non financial incentive could not constantly keep worker to work hard. It is therefore important to keep a balance between non-financial and financial incentives. (DHMT member, 36 years, district headquarter)

#### Team-based versus individual incentive

There was a consensus that financial incentive should be given to the entire team. As commented by one district manager:

I prefer the team-based incentive, as it is the team that wins. Rewarding the entire team will avoid frustrations and promotes transparency. (ZT, male district manager, 39 years, district headquarter)

This statement was also supported by one of the primary health centre (PHC) workers:

I think that rewarding the team is much better. Providing individual reward could pose a problem among the workers, as it is always difficult to define criteria for individual performance assessment. Individual incentive may cause frustration among the others. Workers will be grateful that their health facility is rewarded and the reward will proudly hang out on the wall. (Female midwife, 29 years, PO, PHC)

#### Typology of financial and non-financial incentives to be included in the PBI system

The most cited financial incentives were: 1) salary increase; 2) extra duty pay; 3) bonuses from user fees; and 4) per diem during trainings.

For non-financial incentive, the most cited were: 1) regular training; 2) well-equipped working environment; 3) recognition of work achieved; and 4) good accommodation.

### Existing health facilities revenues to be used for incentive funds

The health workers suggested a number of sources for inclusion in the incentive package. The most frequently cited sources were: 1) revenues from users’ fees; 2) benefits from drug sales; 3) management committee funds; and 4) other incomes generated by the health centre. However, this should be applied in accordance with the national policies. Indeed, at district level, a maximum of 20% of the total health facility income can be redistributed as bonuses among practitioners.

#### Performance indicators

Health professionals suggested various performance indicators related to: 1) maternal; 2) neonatal; and 3) facility management to be considered for incentive allocation. There was a convergence of opinion across categories of respondents. However, the final list of key indicators was adopted during a meeting that convened health workers and other stakeholders (policymakers and regional health managers). The suggested indicators fit into the set of indicators, which are routinely collected through the national health information system and used for performance assessment. The suggested indicators are summarized in Table 3. The same indicators were used to assess both the baseline performance and the performance after intervention.

**Table 3:** Performance indicators suggested by the health workers

Performance indicators under health worker control	Performance indicators out of health worker control
Antenatal care	
– Proportion of pregnancy at risk referred over those at risk detected – Women receiving counselling for safe sex – Number of women where basic biological exams were performed (glucose, syphilis, albumin)	– Antenatal care visits coverage – Tetanus immunization coverage of pregnant women – Iron supply for pregnant women – Intermittent preventive treatment for malaria prophylaxis for pregnant women
Prevention of mother-to-child HIV transmission (PMTCT)	
– Pregnant women who received HIV counselling – Pregnant woman tested positive for HIV and register in the active file	– Adherence rate to HIV testing for pregnant women – Proportion of positive pregnant women receiving complete ART treatment for 3 months among the active file pregnant woman more
Deliveries/postpartum/family planning	
– Women completely screened before discharge from the hospital – Contraceptive prevalence rate	– Proportion of births attended by skilled health personnel – Postpartum care (0–42) days coverage
Newborn care	
– Proportion of newborn with complete measurements – Newborn fully immunized (BCG, polio, DPT, measles, yellow fever, hepatitis, meningitis) – Proportion of babies who receive eye prophylaxis care within 1 h of birth – Timely initiation of breastfeeding	– Proportion of child bearing women who know newborn danger signs – Proportion of under 1 year children attended healthy child care unit
Drugs/consumables availability/management	
– Number of staff meeting performed over those planned – Use of diagnosis and treatment guide for patient management	– Availability of 10 essentials drugs – Availability of minimum equipment for delivery – Availability of diagnosis and treatment guide

## Discussion

In spite of the tremendous experiences of PBF implemented in LMIC, little is known about schemes that better fit the providers’ perspectives. Our paper provides an insight into how to develop an incentive scheme that primarily builds on districts’ own resources. This study highlights the importance of exploring workers’ preferences when designing health interventions. We found that the majority of healthcare providers and managers (85%) were in favour of a financial incentive scheme that rewarded the team. The fact that most workers opted for financial incentives instead of non-financial incentives can be explained by the overall lower levels of salaries served to health workers and the lack of other financial opportunities in remote areas. This was earlier noted by Bocoum et al. (43).

### Workers’ views and preference for financial incentive

The study revealed a high level of interest from healthcare providers in a PBI scheme in which they were fully involved at each step of the development. There was a clear preference for a scheme whereby monetary incentives are provided. To ensure sustainability, workers were supportive of the incentive payment scheme based on districts’ own resources.

From the literature, workers’ preference for financial incentive schemes was documented with Chandler for motivation and money in Tanzania (44). However, schemes may vary across countries depending on the health system as well as socio-demographic, economic, and cultural backgrounds. In Pakistan, PBI was welcomed although there was a general consensus amongst the facility staff that the incentives were not sufficient (23).

A similar study conducted in Nouna on provider payment methods and health worker motivation in CBHI found different results (45). Although there was enthusiasm about this scheme, the qualitative in-depth interviews identified that insufficient levels of capitation payments, the infrequent schedule of capitation payment, and lack of a payment mechanism for reimbursing service fees were perceived as significant sources of health worker dissatisfaction and loss of work-related motivation.

Our results are in line with that of the previous studies, which reported health workers’ preference for schemes that include financial incentive as they offer a great opportunity to achieve more health outcomes by increasing the level of commitment of workers (15, 46). The nature of incentives favoured by health workers and managers was quite comparable and included, salary increases, bonuses, and per diems. These suggested incentives are consistent with those described by (42, 47). Despite contextual difference with the PBF scheme in Rwanda, a randomized control trial of PBF scheme based on a financial incentive scheme was appreciated by workers and yielded an increase in institutional delivery by 23% and preventive service among children under age 5 by 25–50% (19).

Notwithstanding their positive effects, most PBF experiences in LMIC have been criticized (23, 36). They fail to involve health workers in its conceptualization as in Rwanda (18, 19). In Tanzania's PBF scheme, funded by the Catholic Organization for Relief and Development Aid, workers expressed concerns about how the PBF targets were set (28). In Pakistan's PBI scheme, many of the staff were not aware of the details of how the incentives were calculated (16). However, researchers should be aware of these common pitfalls in designing provider-oriented schemes. Involving the providers at the start of the process could enhance their sense of responsibility and ensure their full participation and acceptance at the end.

### Workers’ preference for non-financial incentive schemes

Although our study shows a preference by workers for financial rewards, it is worth considering the viewpoints of the few respondents suggesting that monetary incentive schemes need to be accompanied by a non-monetary incentive schemes as well. Such aspects (48) should include staff development (i.e. training, adequate equipment, supportive supervision, and other recognitions). A study on the role of non-financial incentives and human resource management tools shows that non-financial incentives play an important role as a motivator for health professionals and helps to increase their performance (48).

According to Ellis, cited by Miller and Babiarz (49), using performance incentives to increase providers’ efforts necessarily requires assumptions about what motivates providers. Human motives are complex and other factors such as professional recognition and the esteem of colleagues, pride in one's work, opportunities for professional advancement, etc. undoubtedly play a role. Moreover, although pay-for-performance contracts do strengthen extrinsic incentives, intrinsic motivation is commonly thought to be an important determinant of providers’ effort as well.

### Team-based versus individual-based incentives

Most respondents were in favour of team-based incentives. One of the reasons is probably the difficulty involved in setting individual-performance assessment criteria. However, there is mixed response on the merits. On one hand, it is suggested that rewarding health workers for their own individual performance may create disincentive for teamwork or cooperation (49). On the other hand, when staff share rewards for achieving outcomes, for which they are jointly responsible, this can serve to strengthen the team spirit (50) or may create incentives for free-riding because individual do not bear the full cost of shirking (51).

In the Pakistan PBI scheme, the most common arrangement was for incentives to be paid to facilities (16), which concur with our findings. Similar findings of preference for team-based incentives were reported in Ghana and Tanzania (42).

However, although teamwork has been shown to improve clinical outcomes and provider satisfaction, discussion of how best to incentivize medical teams has been limited (52, 53).

### Performance indicators to be used for rewards

Selecting key performance indicators, on which to base health workers’ performance assessment is essential. This was noted by Miller and Babiarz (49) for whom an alternative for most of the scheme is to reward the use of healthcare service, particularly those that are relatively sensitive to provider effort. Our study showed high interest on maternal and new-born care indicators, including prenatal care visits, institutional deliveries, and immunization coverage. In the present study, the respondents identified overall 28 indicators to measure their performance. In Rwanda, 14 indicators have been identified related to prenatal care visits, immunization for mothers and children, institutional deliveries, and human immunodeficiency virus testing (18, 19).

### Designing an effective incentive scheme

Designing an incentive scheme through a participatory process yielded high interest from health workers. This was recognized as fundamental by Witter from the stakeholders’ interview in the success and adherence of workers on the PBF scheme (16) who highlighted the need to strike a balance between financial and non-financial incentives.

There was also concern that the use of financial incentives may lead to demoralization (22), reductions in intrinsic motivation (54), and decline in the quality of the health workforce if the financial incentive selects against intrinsically motivated healthcare workers (20, 54).

Thus, a mixed approach was useful not only to better estimate the degree of acceptance/rejection of the proposed incentive scheme, but also to better understand workers’ perceptions and the implications of their choices. Furthermore, providing incentives to workers may also be perceived as a means to enhance their level of motivation.

### Description of dissemination and policy development process

The results of this study were first shared in 2010 with all stakeholders during a meeting at the district level. This experience was fundamental in the scheme being adopted by the health workers and the project implementation from 2011. This study has paved the way for the reform in health system financing in Burkina Faso. Nouna district was also involved in the World Bank RBF scheme discussion as CBHI was already implemented. Moreover, this scheme falls into the national strategic plan for the implementation of PBF, which started in 2014. Particularly, this experience benefited the NHD, which recently was selected as PBF plus CBHI interventional district in 2014 in another project funded by the World Bank.

### Value added and policy implication

This paper is innovative in the way that it explores healthcare providers’ views about alternative payment for PBF, which is built upon district resources. The approach adopted showed that it is possible to develop and contextualize a locally adapted PBI scheme with beneficiaries. This paper may serve as a road map to guide policymakers and health managers in developing and implementing sustainable PBI schemes.

### Limitations of the study

The scope of the study was limited to one district in Burkina Faso and could have underestimated individual factors related to acceptance of the proposed scheme, useful to scale up the initiative at the national level. The data were collected with data collectors without any medical background. This could have limited the quality of data collected. In addition, the back translation from French to English may have distorted or lost some information.

## Conclusions

PBI schemes funded through health facilities’ own resources are among the newly proposed schemes that complement traditional donor-funded schemes. However, the implementation of such schemes remains still challenging. The study showed that a participatory approach in designing incentive schemes in which all stakeholders are involved at each step of development was most likely to yield high acceptability among funders as well as beneficiaries. Furthermore, the study raised much enthusiasm among health workers as they were centrally involved at each development stage of the scheme.

## Key messages

- Performance-based incentive (PBI) schemes are widely implemented in developing countries supported mainly by foreign donors. Schemes based on local resources are scarce.
- We proposed a PBI scheme wherein health workers were strongly involved at each development stage.
- This incentive scheme, designed using a participatory approach involving health workers and others stakeholders, was valued and yielded a high acceptance rate.
- The approach helped strengthen the staff participation in the local health governance and paved the way forward for further incentive policy adoption taking into account the local context.

## Authors' contributions

MY: Conception and design of the study, analysis and interpretation of data, drafting the paper, and accepting accountability for all aspects of the work; ED: conception and design of the study, analysis and interpretation of data, drafting the paper, and approving the final version of the paper; MK: analysis and interpretation of data, drafting the paper, and approving the final version of the paper; AS: conception and design of the study, drafting the paper, and substantially revising the paper; RS: drafting the paper, approving the final version to be published, and accepting accountability for all aspects of the work; SL: conception and design of the study, analysis and interpretation of data, drafting the paper, and approving the final version to be published.

## Conflict of interest and funding

The authors declare that they have no competing interests.

## Supplementary Material

Health worker preferences for performance-based payment schemes in a rural health district in Burkina Faso
