# Supplementary material for: Health worker preferences for performance-based payment schemes in a rural health district in Burkina Faso
Source: Glob Health Action. 2016 Jan 5;9:10.3402/gha.v9.29103. doi: 10.3402/gha.v9.29103 (PMC4703797; doi:10.3402/gha.v9.29103)
Supplement: Health worker preferences for performance-based payment schemes in a rural health district in Burkina Faso [file GHA-9-29103-s001.doc]

Supplementary Table 1: Key terms definitions

| **Terms** | **Definition** |
| --- | --- |
| Performance-based incentive (PBI) | Any program that rewards the delivery of one or more outputs or outcomes by one or more incentives, financial or otherwise, upon verification that the agreed upon result has actually been delivered (21) |
| Performance-based financing (PBF) or pay-for-performance (P4P) | Involves the “transfer of money or material goods conditional on taking a measurable action or achieving a predetermined performance target”(18) |
| Results-based financing (RBF) | A cash payment or non monetary transfer made to a national or sub-national government, manager, provider, payer or consumer of health services after predefined results have been attained and verified (55) |
| Provider-based financing | Incentives are paid to the provider based on a set of performance targets or indicators, which are mostly linked to the number of beneficiaries (56). |
| Demand- based financing | Payment, which focuses only on consumer. It’s the direct link between the payment of incentives and the intended beneficiary as well as the desired result (56). |
| Financial incentive | Involves the transfer of monetary values such us salaries, pensions, bonuses, allowances, loans, etc.(48). |
| Non financial incentive | Includes work autonomy, flexible hours and scheduling, recognition of work, coaching and mentoring structures, support for career development etc.(Mathauer & Imhoff, 2006). |

**Supplementary Table 2**: Acceptability and preference of health workers for incentive scheme and level of financial contribution (n=94)

| **Variables** | **Agree** | **Desagree** | **Total** | **95% CI** | **P-value** |
| --- | --- | --- | --- | --- | --- |
| **Accept internal incentive system** | 80 (85%) | 14 (15%) | 94 (100) | [71.91; 88.08] | P<0.001 |
| **Financial versus non- financial** | 76 (95%) | 4 (5%) | 80 (100%) | [66.64; 85.36] | P<0.001 |
| **Team-based versus individual incentive system** | 77(96%) | 3 (4%) | 80 (100%) | [67.78; 86.22] | P<0.001 |
| **Proportion of workers in favour of 10% contribution** | 37(46%) | 43(54%) | 80(100%) | 24.42; 47.58] | P=0.43 |
| **Proportion of workers in favour of 20% contribution** | 19 (24%) | 61(76%) | 80 (100%) | [10.40; -27.60] | P<0.001 |

**Legend:** CI: confidence interval; PBI: performance-based incentive.
